# Supplementary material for: Striatal lateral inhibition regulates action selection in a mouse model of levodopa-induced dyskinesia
Source: bioRxiv. 2024 Nov 1:2024.10.11.617939. Originally published 2024 Oct 12. Preprint. [Version 2] doi: 10.1101/2024.10.11.617939 (PMC11482940; doi:10.1101/2024.10.11.617939)
Supplement: Supplement 1 — Figure S1. MSN-MSN lateral connections are mediated by GABAA receptors. Figure S2. Unilateral depletion of dopamine causes rotational bias. Figure S3. D1-D2 synaptic responses are not remodeled in parkinsonian animals and parkinsonian animals chronically treated with levodopa. Figure S4. Chemogenetic inhibition of striatal lateral connections originating from D2-MSNs is not sufficient to cause dyskinesia without levodopa. Table S1. Statistical table. [file media-1.pdf]

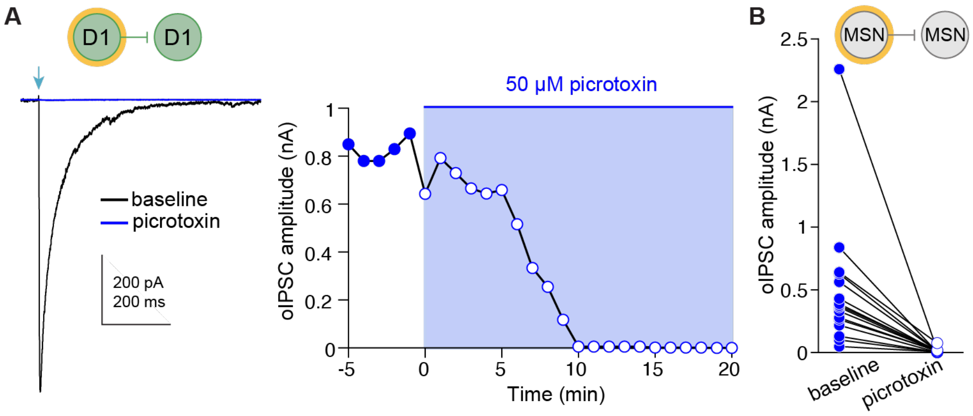

Figure S1. *MSN-MSN lateral connections are mediated by $GABA_A$ receptors.*

(A) (*Left*) Representative example of D1-D1 oIPSC before and after application of 50 µM picrotoxin. (*Right*) Time course of the effect of picrotoxin on oIPSC amplitude.

(B) Summary of change in oIPSC amplitude in response to picrotoxin. MSN-MSN connection types are pooled. N = 13, n = 16. WSR: $p < 0.0001$. N = animals; n = cells. Data shown as mean ± SEM.

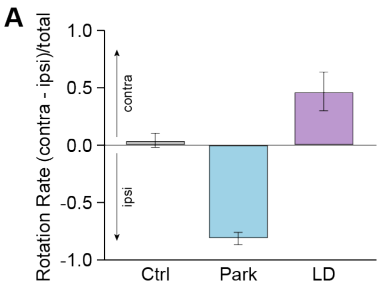

Figure S2. *Unilateral depletion of dopamine causes rotational bias.*

(A) Rotation rate in the open field over 10 minutes control (Ctrl), parkinsonian (Park and LD OFF medication), and parkinsonian mice treated with levodopa (LD ON medication). Rotation rate is calculated as number of ipsilesional rotations subtracted from the number of contralesional rotations divided by the total number of rotations. Ctrl: N = 9, Park: N = 14, LD: N = 8. N = animals. Data shown as mean ± SEM.

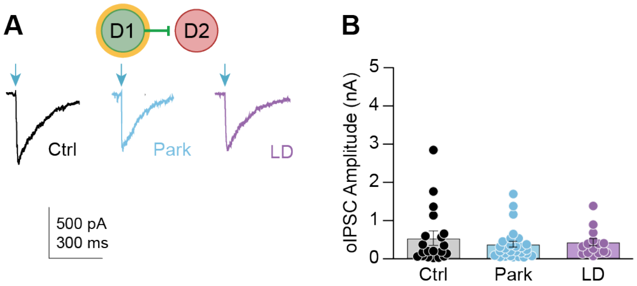

Figure S3. *D1-D2 synaptic responses do not differ across experimental groups.*

- **(A)** Representative D1-D2 oIPSCs in animals belonging to Ctrl (*left*), Park (*middle*), and LD (*right*) groups.
- **(B)** Average D1-D2 oIPSC amplitude in response to 1 mW optical stimulation for Ctrl (N = 9, n = 20), Park (N = 4, n = 30), and LD (N = 9, n = 16) groups. KW: $p = 0.64$. Each overlaid dot represents one cell. N = animals; n = cells. Data shown as mean ± SEM.

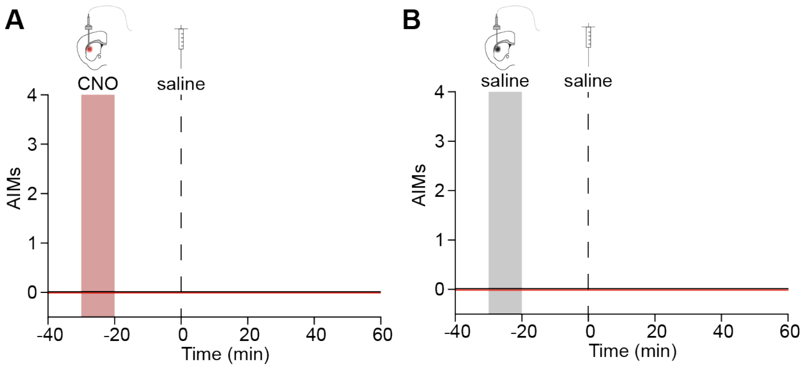

Figure S4. *Chemogenetic inhibition of striatal lateral connections originating from D2-MSNs is not sufficient to cause dyskinesia without levodopa.*

(A) – **(B)** No dyskinesia was observed following local striatal infusion of CNO (A) or saline (B) and IP injection of a low-dose of levodopa hM4D(G_i): N = 14, mCherry: N = 12. N = animals. Data shown as mean ± SEM.

Key Experiments	Figure	Type of Comparison	Statistical Test	n (cells)	N (animals)	Comparison values (± SEM) & p-value
**Ctrl oIPSC amplitude, 1 mW (all groups)**	1G	Between-group	KW	D2-D1: 41; D1-D1: 13; D1-D2: 20; D2-D2: 8	D2-D1: 12; D1-D1: 4; D1-D2: 9; D2-D2: 5	X^2 = 19.1; p = 0.0003
**Ctrl oIPSC amplitude (D2-D1 vs D1-D1)**	1G	Between-group	Dunn's	D2-D1: 41; D1-D1: 13	D2-D1: 12; D1-D1: 4	D2-D1 vs D1-D1, p = 0.0223
**Ctrl oIPSC amplitude (D2-D1 vs D1-D2)**	1G	Between-group	Dunn's	D2-D1: 41; D1-D2: 20	D2-D1: 12; D1-D2: 9	D2-D1 vs D1-D2, p = 0.0034
**Ctrl oIPSC amplitude (D2-D1 vs D2-D2)**	1G	Between-group	Dunn's	D2-D1: 41; D2-D2: 8	D2-D1: 12; D2-D2: 5	D2-D1 vs D2-D2, p = 0.0314
**Ctrl oIPSC amplitude (D1-D1 vs D1-D2)**	1G	Between-group	Dunn's	D1-D1: 13; D1-D2: 20	D1-D1: 4; D1-D2: 9	D1-D1 vs D1-D2, p > 0.9999
**Ctrl oIPSC amplitude (D1-D1 vs D2-D2)**	1G	Between-group	Dunn's	D1-D1: 13; D2-D2: 8	D1-D1: 4; D2-D2: 5	D1-D1- vs D2-D2, p > 0.9999
**Ctrl oIPSC amplitude (D1-D2 vs D2-D2)**	1G	Between-group	Dunn's	D1-D2: 20; D2-D2: 8	D1-D2: 9; D2-D2: 5	D1-D2 vs D2-D2, p > 0.9999
**MSN-MSN oIPSC amplitude after picrotoxin**	S1	Within-cell	WSR	16	13	p < 0.0001
**D1-D1 oIPSC amplitude, 1 mW (all groups)**	2G	Between-group	KW	Ctrl: 13; Park: 15; LD: 12	Ctrl: 4; Park: 6; LD: 7	X^2 = 1.358; p = 0.5071
**D1-D1 oIPSC amplitude (Ctrl vs Park)**	2G	Between-group	Dunn's	Ctrl: 13; Park: 15	Ctrl: 4; Park: 6	Ctrl vs Park, p > 0.9999
**D1-D1 oIPSC amplitude (Ctrl vs LD)**	2G	Between-group	Dunn's	Ctrl: 13; LD: 12	Ctrl: 4; LD: 7	Ctrl vs Park, p > 0.9999
**D1-D1 oIPSC amplitude (Park vs LD)**	2G	Between-group	Dunn's	Park: 15; LD: 12	Park: 6; LD: 7	Park vs LD, p = 0.8036
**D2-D1 oIPSC amplitude, 1 mW (all groups)**	2I	Between-group	KW	Ctrl: 41; Park: 24; LD: 32	Ctrl: 12; Park: 6; LD: 8	X^2 = 14.68; p = 0.0006
**D2-D1 oIPSC amplitude (Ctrl vs Park)**	2I	Between-group	Dunn's	Ctrl: 41; Park: 24	Ctrl: 12; Park: 6	Ctrl vs Park, p = 0.0288
**D2-D1 oIPSC amplitude (Ctrl vs LD)**	2I	Between-group	Dunn's	Ctrl: 41; LD: 31	Ctrl: 12; LD: 8	Ctrl vs LD, p = 0.3685
**D2-D1 oIPSC amplitude (Park vs LD)**	2I	Between-group	Dunn's	Park: 24; LD: 32	Park: 6; LD: 8	Park vs LD, p = 0.0004
**D1-D2 oIPSC amplitude, 1mW (all groups)**	S3	Between-group	KW	Ctrl: 20; Park: 30; LD: 16	Ctrl: 9; Park: 4, LD: 9	X^2 = 0.8926; p = 0.64
**D1-D2 oIPSC amplitude (Ctrl vs Park)**	S3	Between-group	Dunn's	Ctrl: 20; Park: 30	Ctrl: 9; Park: 4	Ctrl vs Park, p > 0.9999
**D1-D2 oIPSC amplitude (Ctrl vs LD)**	S3	Between-group	Dunn's	Ctrl: 20; LD: 16	Ctrl: 9; LD: 9	Ctrl vs LD, p > 0.9999
**D1-D2 oIPSC amplitude (Park vs LD)**	S3	Between-group	Dunn's	Park: 30; LD: 16	Park: 4; LD: 9	Park vs LD, p > 0.9999
**Norm. D2-D1 oIPSC after quinpirole (all groups)**	3D	Between-group	KW	Ctrl: 11; Park: 7; LD: 9	Ctrl: 6; Park: 4; LD: 3	X^2 = 3.165; p = 0.2055
**Norm D2-D1 oIPSC after quinpirole (Ctrl vs Park)**	3D	Between-group	Dunn's	Ctrl: 11; Park: 7	Ctrl: 6; Park: 4	Ctrl vs Park, p > 0.9999
**Norm D2-D1 oIPSC after quinpirole (Ctrl vs LD)**	3D	Between-group	Dunn's	Ctrl: 11; LD: 9	Ctrl: 6; LD: 3	Ctrl vs LD, p = 0.3296
**Norm D2-D1 oIPSC after quinpirole (Park vs LD)**	3D	Between-group	Dunn's	Park: 7; LD: 9	Park: 4; LD: 3	Park vs LD, p = 0.4026
**Ctrl D2-D1 oIPSC amplitude after quinpirole**	3D	Within-Cell	WSR	11	6	p = 0.001

**Park D2-D1 oIPSC amplitude after quinpirole**	3D	Within-Cell	WSR	7	4	p = 0.0156
**LD D2-D1 oIPSC amplitude after quinpirole**	3D	Within-Cell	WSR	9	3	p = 0.0039
**D2-D1 oIPSC amplitude (w/ hM4Di) after CNO**	S4C	Within-Cell	WSR	6	2	p = 0.0312
**AIMs: CNO infusion + IP levodopa (hM4Di vs mCherry)**	4E	Between-group	2-way RM ANOVA	NA	hM4Di: 14, mCherry: 12	p < 0.001
**AIMS: IP CNO + IP levodopa (hM4Di vs mCherry)**	4I	Between-group	2-way RM ANOVA	NA	hM4Di: 14, mCherry: 12	p = 0.0059

Table S1. *Statistical table.*

Each row in the table provides key information regarding a statistical comparison made in the manuscript, including the figure, statistical test, n/N, p-value, and planned sample size based on power calculations. Abbreviations: KW (Kruskal-Walis Test), Dunn's (Dunn's Multiple Comparisons Test), WSR (Wilcoxon Signed Rank), 2-way RM ANOVA (2-way repeated measures analysis of variance). *p-value corrected for multiple comparisons.

Key resources table

REAGENT or RESOURCE	SOURCE	IDENTIFIER
Antibodies		
Rabbit anti-Tyrosine Hydroxylase	Pel-Freez Biologicals	RRID: AB_2617184; Cat #P40101-150
Alexa Flour 488 - Donkey Anti-Rabbit	Jackson ImmunoResearch	RRID: AB_2340619; Cat#711-546-152
Alexa Flour 647 - Donkey Anti-Rabbit	Jackson ImmunoResearch	RRID: AB_2340625; Cat #711-606-152
Bacterial and virus strains		
AAV5-hSyn-DIO-mCherry	UNC	RRID: Addgene_50459; Lot# AV4634C
AAV5-hSyn-DIO-hM4D(Gi)-mCherry	Addgene	RRID: Addgene_44362; Lot #v120251
AAV5-EF1a-DIO-hCHR2(H134R)-eYFP-wpre-hGH	Penn	RRID: Addgene_20298; Lot# CS1046
AAV5-EF1a-DIO-hCHR2(H134R)-eYFP-wpre-hGH	Penn	RRID: Addgene_20298; Lot# CS0706
Biological samples		
Chemicals, peptides, and recombinant proteins		
Picrotoxin	Sigma-Aldrich	P1675
Quinpirole hydrochloride	Tocris	1061
Lidocaine N-ethyl chloride	Sigma-Aldrich	L1663
6-Hydroxydopamine hydrobromide	Sigma-Aldrich	162957
Guanosine 50 -triphosphate sodium salt hydrate	Sigma-Aldrich	G8877
Adenosine 50 -triphosphate magnesium salt	Sigma-Aldrich	A9187
Desipramine hydrochloride	Sigma-Aldrich	D3900
Cesium methanesulfanate	Sigma-Aldrich	C1426
Potassium methanesulfanate	Sigma-Aldrich	83000
Cesium chloride	Sigma-Aldrich	C3139
Clozapine-N-Oxide	Tocris	6329
FM4-64	ThermoFischer Scientific	T13320
Levodopa (3,4-Dihydroxy-L-phenylalanine)	Sigma-Aldrich	D9628
Beserazide	Sigma-Aldrich	B7283
Critical commercial assays		

VECTASHIELD Antifade Mounting Medium	Vector Laboratories	RRID: AB_2336789; Cat#H-1000
Deposited data		
Experimental models: Cell lines		
Experimental models: Organisms/strains		
Mouse: B6.FVB(Cg)-Tg (Drd1- cre) EY217Gsat/ Mmucd	MMRRC	RRID: MMRRC_034258-UCD
Mouse: B6.FVB(Cg)-Tg (Adora2acre) KG139Gsat/ Mmucd	MMRRC	RRID: MMRRC:036158-UCD
Mouse: STOCK Tg(Drd2- EGFP)S118Gsat/ Mmnc Mus musculus	MMRRC	RRID: MMRRC_000230-UNC
Mouse: Stock Tg(Drd2-EGFP)S118Gsat/Mmnc Mus Musculus	MMRRC	RRID: MMRRC_000230-UNC
Mouse: B6.Cg-Tg(Drd1a-tdTomato)6Calak/J Mus musculus	The Jackson Laboratory	RRID: MSR_JAX:016204
Mouse: WT: C57BL/6J	The Jackson Laboratory	RRID: IMSR_JAX:000664
Oligonucleotides		
Recombinant DNA		
Software and algorithms		

Igor Pro	Wavemetrics	RRID: SCR_00325; http://www.wavemetrics.com/products/igorpro/ igorpro.htm
mafPC (software package for use with IgorPro)	Xu-Friedman Lab	https://www.xufriedman.org/mafpc
ImageJ/FIJI	NIH	RRID: SCR_003070; https://imagej.nih.gov/ij/
EthoVision XT	Noldus	RRID: SCR_000441; http://www.noldus.com/animal-behavior-research/ products/ethovision-xt
Adobe Illustrator	Adobe	RRID: SCR_010279; https://www.adobe.com/products/illustrator.html
Axon MultiClamp Commander	Axon	RRID: SCR_018455; http://mdc.custhelp.com/ app/answers/detail/a_id/18877/axon%E2%84%A2-multi
GraphPad Prism 10	GraphPad	RRID: SCR_002798; http://www.graphpad.com/features
Other		
Guide cannula	Protech International, Inc.	C315GS-5/SP; cut 5 mm below pedestal; https://protechinternational.com/products/26-gauge-short-pedestal-guide-c315gs-5-spc
Dummy cannula	Protech International, Inc.	C315DCS-5/SPC DUMMY (SM) .008/.2MM; Fit 5mm Guide with 0.5mm Projection; https://protechinternational.com/products/dummy-cannula-c315dcs-5-spc
Connector cannula	Protech International, Inc.	Cat#C313C; https://protechinternational.com/products/connector-cannula-c313c-with-no-spring

Cannula tubing	Protech International, Inc.	Cat#C313CT; https://protechinternational.com/products/cannula-tubing-c313ct-pkg
Single Channel Temperature Controller	Warner Instruments	Cat#TC-324C
MINIPULS 3 Peristaltic Pumps	Gilson	Cat#F155008
X-Cite 120LED Boost	Excelitas	Cat#010-00326R
ITC-18 16-bit Multi-Channel Data Acquisition Interface	Heka	RRID: SCR_023164
Multiclamp 700B Microelectrode Amplifier	Molecular Devices	RRID: SCR_018455
Monochrome Industrial Camera	ImagingSource	Cat#: DMK33UX273
Hamilton 11 Plus Syringe Pump	Harvard Apparatus	Cat#:70-2209
Micro4 Micro Pump	WPI	Cat#: UMP3
Micro4 Pump Controller	WPI	Cat#: UMP4
Nanofil 33 GA Blunt Needle	WPI	Cat#: NF33BL
Nanofil Syringe 10 µL	WPI	Cat#: NANOFIL
